# To Decipher the *Mycoplasma hominis* Proteins Targeting into the Endoplasmic Reticulum and Their Implications in Prostate Cancer Etiology Using Next-Generation Sequencing Data

**DOI:** 10.3390/molecules23050994

**Published:** 2018-04-24

**Authors:** Mohammed Zakariah, Shahanavaj Khan, Anis Ahmad Chaudhary, Christian Rolfo, Mohamed Maher Ben Ismail, Yousef Ajami Alotaibi

**Affiliations:** 1 Research Center, College of Computer and Information Science, King Saud University, Riyadh 11451, Saudi Arabia; mzakariah@ksu.edu.sa; 2 Nanomedicine & Biotechnology Research Unit, Department of Pharmaceutics, College of Pharmacy, P.O. Box 2457, King Saud University, Riyadh 11451, Saudi Arabia; 3 Department of Bioscience, Shri Ram Group of College (SRGC), Muzaffarnagar 251002, UP, India; 4 Department of Pharmacology, College of Medicine, Al-Imam Mohammad Ibn Saud Islamic University, Riyadh 11451, Saudi Arabia; anis.chaudhary@gmail.com; 5 Phase I-Early Clinical Trials Unit, Oncology Department, Antwerp University Hospital, “Centre for Oncological Research (CORE)”, 2650 Edegem, Belgium; Christian.Rolfo@uza.be; 6 Computer Science Department, College of Computer and Information Sciences, King Saud University, Riyadh 11451, Saudi Arabia; mbenismail@ksu.edu.sa; (M.M.B.I.); yaalotaibi@ksu.edu.sa (Y.A.A.)

**Keywords:** prostate cancer, *Mycoplasma hominis*, endoplasmic reticulum, systems biology, protein targeting

## Abstract

Cancer was initially considered a genetic disease. However, recent studies have revealed the connection between bacterial infections and growth of different types of cancer. The enteroinvasive strain of *Mycoplasma hominis* alters the normal behavior of host cells that may result in the growth of prostate cancer. The role of *M. hominis* in the growth and development of prostate cancer still remains unclear. The infection may regulate several factors that influence prostate cancer growth in susceptible individuals. The aim of this study was to predict *M. hominis* proteins targeted into the endoplasmic reticulum (ER) of the host cell, and their potential role in the induction of prostate cancer. From the whole proteome of *M. hominis*, 19 proteins were predicted to be targeted into the ER of host cells. The results of our study predict that several proteins of *M. hominis* may be targeted to the host cell ER, and possibly alter the normal pattern of protein folding. These predicted proteins can modify the normal function of the host cell. Thus, the intercellular infection of *M. hominis* in host cells may serve as a potential factor in prostate cancer etiology.

## 1. Introduction

Bacterial infection is recognized to play a significant role in the progression and advancement of various forms of cancers, including prostate, lung, gastric, and colon cancer [1,2,3]. Recent study showed that prostate gland restrains a plethora of different strains of bacteria [4]. Bacterial dysbiosis, inflammation, and other factors are associated with the growth of prostate cancer, although the exact mechanisms involved in growth of cancer due to bacterial infection are not very clear. Mycoplasmas are bacteria that lack cell walls, and are causative agents of various diseases related to respiratory and urogenital tract among humans [5,6]. The dominant types of mycoplasmas found in the urogenital system of humans include *Mycoplasma genitalium*, *Ureaplasma urealyticum*, and *M. hominis*. The relationship between mycoplasmas and the human population was first detected in the 1960s [7]. Different studies have highlighted the connection between *M. hominis* infection and prostate cancer advancement [8,9,10,11]. This association was further supported by numerous studies on *M. hominis* infection and higher classification of prostate cancer. The cell cycle signal cascade, including DNA repair mechanisms and apoptosis, may be altered following mycoplasma infection [8]. Although a meta-analysis report has suggested a suspicious role of *M. hominis* in the growth of prostate cancer [12], various recent studies instead confirmed the involvement of different bacteria in the progression of different types of cancer [13,14,15]. Pathogenic bacteria and their subcellular constituents interact with several types of host cell receptors that may change the expression of various genes. These enigmatic alterations may affect the normal control and regulation of host cells [8,16]. Chronic inflammation and chronic infections (or both) are the cause of 20% of different forms of cancers in humans.

Induction of *pro*-*inflammatory cytokines* and reactive *oxygen species*, regulated by *chronic inflammation*, may promote nitration and chlorination of nucleic acids and proteins. *M. hominis* infection serves as a factor promoting the growth and development of prostate cancer, but several other causes are linked with the growth of prostate cancer [17,18,19]. Apart from chronic inflammation and mutations, different cyclomodulins have been associated with the growth of prostate cancer by the disturbance of homeostasis in *M. hominis*-infected cells. Some specific strains of bacteria have ability to produce different toxins known as cyclomodulins, that interfere in the host cell cycle, which suggests a potential association of different pathogenic bacteria with different type of cancers [20]. It has supposed that cyclomodulins have the capability to affect the usual growth cycle of the host cell, and are expected to grow as etiological aspects for *M. hominis*-mediated prostate cancer [8]. *M. hominis* strain is considered a usual Gram-negative pathogen. It is known to multiply and inhabit intracellularly during the progression of prostate cancer [21,22]. As *M. hominis* is colonized in the urogenital tract that comprises the prostate, *M. hominis* infection leads to some precise effects in the progression of prostate cancer. The mass collection of genomes for *M. hominis* revealed 715,165 base pairs and a G + C content of 26.94%.

Various strains of different bacteria are involved in the intracellular infection and duplication in specific host cells, wherein bacterial pathogens change the usual functioning of cells through the localization of their own proteins in different components of host cells, such as endoplasmic reticulum (ER), Golgi complex, mitochondria, nucleus, plasma membrane, and cytoplasm [13,21,22]. The complete genome of ATCC 27545 strain of *M. hominis* contains 563 open reading frames (ORFs) and encodes different enzymes and proteins. *M. hominis* has the capacity to naturally undergo intercellular replication, allowing the localization of numerous proteins within the host. The targeted proteins work as component of the host cell proteome. Hence, it is likely that several *M. hominis* proteins may possibly get localized within the host cell, due to the availability of signature sequence and evolutionary relatedness of proteins targeted within the cellular compartments of host cells. ER is an main compartment involved in proper protein folding, post-translation modification, translocation, and regulation of cellular homeostasis [23,24]. The unfolded protein response (UPR) is a highly conserved evolutionarily adaptive response that disrupts the ER physiology. UPR has been shown to be altered by different viruses and plays various roles during bacterial infection [25,26]. Both UPR and ER stress activation are involved in the growth and progression of various types of cancers [27]. The whole proteome of *M. hominis* may disturb the normal behavior of infected host cells and get involved in the development of prostate cancer. The main objective of the current work was to predict the protein localization of *M. hominis* in host cells and evaluate their role in the etiology of prostate cancer. We focused on ER proteins using bioinformatics tools and techniques and explored protein localization of *M. hominis* in the ER of host cells. We investigated the possible implication and relations of *M. hominis* proteome in the etiology of prostate cancer.

## 2. Results

### 2.1. Selection of the Whole Proteome Database of M. hominis

The whole protein sequence of ATCC-27545 strain of *M. hominis* was collected from UniProt, which has maximum proteins (563) with respect to other available strains. The unique selection of UniProt database was associated with its specific characteristics, including opulent, entirely classified, comprehensive, and accurately annotated sequences.

### 2.2. Prediction of the Subcellular Localization by cNLS Mapper

**Figure 1:**
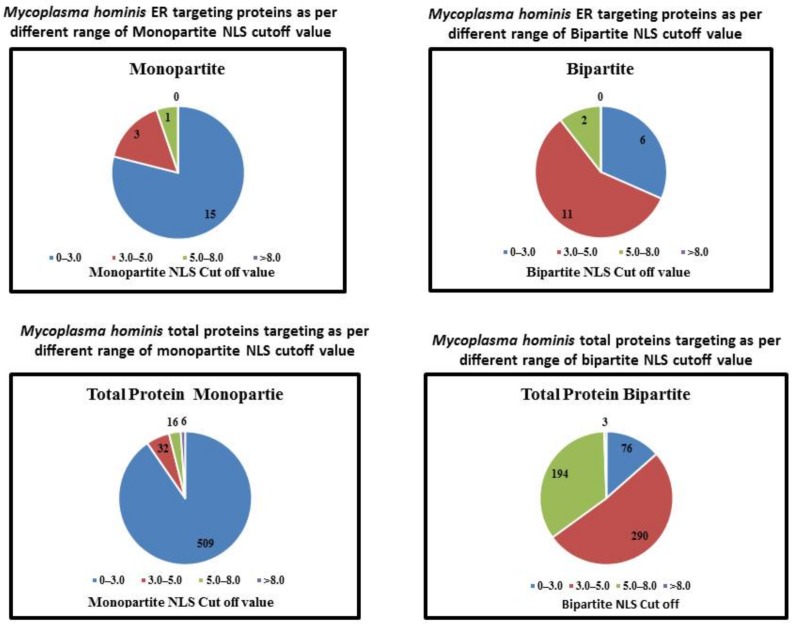
In silico analyses of the *Mycoplasma hominis* proteins localization in endoplasmic reticulum of host cells and their relationship with various NLS values.

Nineteen proteins of *M. hominis* were predicted to be targeted in the ER with different NLSs (Figure 1). Figure 1 shows that among 19 ER targeting proteins, 15, 03, and 01 protein exhibit 0–3.0, 3.0–5.0, and 5.0–8.0 monopartite NLSs cutoff values respectively. Furthermore, the bipartite NLS was observed in 19 proteins and 06, 11, and 02 proteins exhibited 0–3.0, 3.0–5.0, and 5.0–8.0 cutoff values for NLSs, respectively. Different *M. hominis* proteins in a particular host cell may change the usual functioning of the host cell and promote the process of growth and development of cancer. cNLS (classical nuclear localization signals) mapper worked on amino acid sequence patterns, executed by three easy rules according to the NLSs classification [28]; these three rules are principally clusters of K and R basic amino acids and spaces between the clusters. cNLS mapper predicted nuclear localization signals for eukaryotic cells in *M. hominis* proteins. The literature of cNLS mapper has showed that the proteins with cut off value 8–10, 7–8, 3–5, and 1–2, were analyzed as targeted to the nucleus, targeted to both nucleus, partially targeted to nucleus and cytoplasm, and targeted to the cytoplasm, respectively. On the existence of multiple NLS sequence in same proteins, the elevated cut off value was documented.

### 2.3. Prediction of ER Localization in M. hominis Proteins Using Hum-Mploc 3.0

From the whole proteins (563) of *M. hominis*, only 19 were observed to be targeted into the ER of the host cell, as analyzed using Hum-mPLoc 3.0. The increase in cutoff values of NLS in monopartite and bipartite resulted in a decrease in ER localization (except 3.0–5.0 cutoff value of bipartite) (Table 1). Similarly, the increase in the molecular weight resulted in the decrease in the ER protein targeting of *M. hominis* (except for the range of 0–20 kDa). Proteins of 20–40 kDa molecular weights were observed mainly localized in the ER of the host cell (Table 2). Moreover, the outcome of values of isoelectric point (pI) failed to illustrate any constant pattern for ER localization of different proteins of *M. hominis* (Table 3). However, it has demonstrated that the bimodal character (pI and subcellular localization) in bacteria is likely to be a common property of proteomes, and is connected with the requirement of various pI values depending on subcellular protein localization [29].

**Figure 2:**
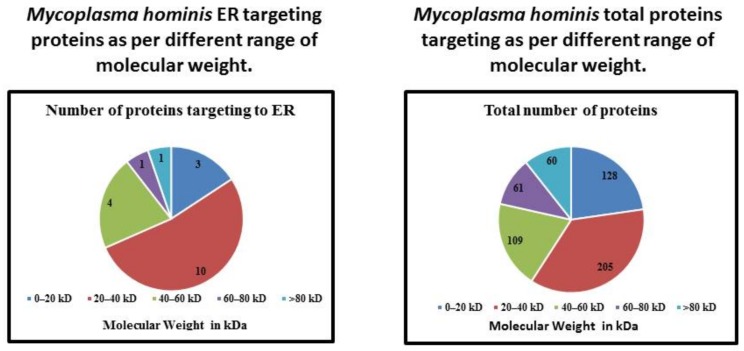
In silico analyses of the *Mycoplasma hominis* proteins localization in endoplasmic reticulum of host cells and their relation to proteins with different ranges of molecular weight.

**Figure 3:**
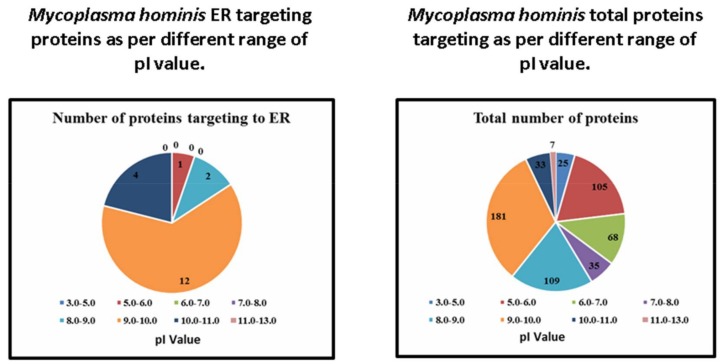
In silico analyses of the *Mycoplasma hominis* proteins localized in endoplasmic reticulum of host cells and their relation to proteins with different ranges of pI values.

The protein localization patterns of *M. hominis* in the ER of the host cell with diverse NLS values are shown in Figure 1. The patterns of protein localization in *M. hominis* for various ranges of molecular weights are shown in Figure 2. In the Figure 3, we illustrate the protein localization patterns of *M. hominis* in the ER of the host cell at various ranges of pI values. Various ER-targeting proteins are thought to be involved in different biochemical pathways. The details of the outcome of the ER-targeting *M. hominis* proteins with different roles are shown in Table 4. Various localized proteins could interfere in the regular growth behavior of host cells, thus leading to the alteration in the regular functioning of the host cell biochemical pathways. We have suggested a possible association of ER-targeting proteins of *M. hominis* in prostate cancer etiology. We have recently predicted the possible impacts of nucleus-, mitochondria-, and cytoplasm-targeting *M. hominis* proteins on the carcinogenesis of prostate cancer [10,30].

## 3. Discussion

Prostate cancer is the frequently identified cancer and sixth leading reason of cancer-related death globally. The frequency of prostate disease has relentlessly expanded in Asian nations, including China, India, and Malaysia [19]. Cancer was initially considered as a genetic disease. Nevertheless, various studies have revealed the association between bacterial infections and progression of different cancer types. The infection may regulate different factors that influence prostate cancer progress in susceptible individuals. It has been reported that protein molecules of host cells face chronic and acute challenges for the maintenance of their integrity [31]. Proteostasis/protein homeostasis allows health and proper growth of eukaryotic cells. The proteostasis depends upon a complex network of proteases molecular, proteins, chaperones, and different regulatory factors [32]. Inadequacies in protein homeostasis or proteostasis direct to several cardiovascular, neurodegenerative, metabolic, and oncological disorders [31]. Different factors, such as chemical toxins, exogenous proteins, drugs, and environmental factors compromise proteostasis, which stimulates proteome stress, and cause different disorders [33,34]. Proteostasis in humans is regulated through quality control (QC) network of approximately 800 different proteins [35,36]. The ER is an important subcellular compartment responsible for the regulation of protein folding, post-translation modification, and protein translocation. Interruption in the environment of ER by pathological agents may cause alterations in glycosylation, DNA damage, nutrient deprivation, oxidative stress, calcium depletion, and energy fluctuation/disturbance, thereby resulting in ER stress and consequent accretion of misfolded or unfolded proteins in the ER lumen. These host cells must overcome perturbations of ER functions and stress for their survival. If ER stress is left unresolved, it may disturb the normal functioning of apoptosis [37]. The disturbance in apoptotic regulatory protein Bcl-2 function results in the increased transcription of p53 unregulated modulator of apoptosis (PUMA), Bcl2-like11 (BIM), BH3-only proteins, and NADPH oxidase activator (NOXA). ER stress promotes the interactions between Bax and PUMA, resulting in the release of cytochrome *c* and apoptosis activation by caspase-dependent modulation of p53 proteins. Many *M. hominis* proteins outmaneuver the host cell machinery, and possibly change the normal behavior of host cells, which may promote the growth of prostate cancer. The involvement of *M. hominis* in the progression of prostate cancer is not yet clear. The rationale of this study was to predict *M. hominis* proteins that are targeted into the ER of the host cell, and assess their potential roles in the growth and progression of prostate cancer.

Of the whole proteome, 19 proteins of *M. hominis* were expected to be targeted to the ER of host cells. These predicted proteins of *M. hominis* may modify the normal function of the host cell. The study of protein targeting into the host cell is very important to detect the progression and development of cancer, especially if the cancer growth is associated with the intracellular bacterial infection. The targeting of different bacterial proteins into host cell compartments, such as cytoplasm, mitochondria, and nucleus, has an important effect on the etiology of different cancer types [14,15,38]. The host cell gets affected by various types of bacterial proteins, which alter the usual development and normal behavior of the host cell. Infection is considered as a possible factor in the progression and development of different types of cancer, especially when it is linked with chronic inflammation that leads to cancer progression in various cases [39,40]. Several novel techniques could be developed to detect and treat cancer based on the study of chronic infection related to cancer and their mechanisms and activities that promote cancer [40]. The potential involvement of infection of *M. hominis* in prostate cancer and its evolution and progression depend on the estimation of its protein targeting into various sections of host cells. The protein-targeting ability of *M. hominis* may lead to several consequences related to prostate cancer etiology. Various *M. hominis* proteins targeted into host cells may disturb the behavior and functioning of infected cells [3,9].

Numerous advanced techniques have been developed for the analysis of protein localization; however, these may be inefficient, owing to their high cost and time-consuming protocols [41]. Several bioinformatics tools have been developed to calculate the subcellular targeting of proteins, thereby offering several advantages for investigational procedures [42,43,44]. The tools developed basically focus on the identity/alignment search or recognition of a particular sequence motif essential for particular protein localization [44]. The research work of protein targeting is also essential to infer the different functions of bacterial proteins within the host cell. The possible role of targeted proteins can be analyzed on the evidence of their relationships with different host proteins, whose role is already revealed. This information will act as a starting point for upcoming wet lab experiments.

Although nuclear-targeting proteins play a crucial role by controlling the normal functioning of host cells, other cell organelle-targeting proteins are also involved in the regulation of the normal behavior of host cells. At present, different predictors are developed for the prediction of specific motifs in protein sequences. Six NLS classes have been classified, wherein nuclear import proteins are transferred through α/β pathway of importin. The well-known dimer of α and β importin is present in the nuclear import receptor, wherein α importin works as a possible adapter protein and binds to cNLS that is identified either twice (bipartite NLS) or once (monopartite NLS), with highly basic stretches of amino acids [45]. The potential activity of NLS sequences changes within the identical class of NLS with altered sequence of NLS [46]. Hence, in the current study we particularly employed NLS mapper predictor in the present study to analyze NLS activity as an alternative to NLS sequence based on the contribution of every residue of amino acid in the NLSs. This could lead to more accurate prediction of results [44]. NLS predictor senses the activity of an NLS as a separate protein sequence, rather than the whole structural sequence of a particular native protein.

Hum-mPLoc 3.0 was used in the current in silico study for the analysis of *M. hominis* proteins targeted into the host subcellular compartment. Hum-mPLoc 3.0 tool is based on different Support Vector Machine (SVMs) method systematized in a decision tree, and predicts 12 different human subcellular localization, including ER, nucleus, mitochondrion, cytoplasm, Golgi apparatus, centriole, cytoskeleton, endosome, peroxisome, lysosome, extracellular region, and plasma membrane [47]. The tRNA threonylcarbamoyladenosine biosynthesis protein TsaE, membrane protein, lipoprotein signal peptidase, 1-acyl-*sn*-glycerol-3-phosphate acyltransferase, prolipoprotein diacylglyceryl transferase, cobalt ABC transporter permease, ComEC/Rec2-related protein, protein translocase subunit SecY, and potassium transporter KtrB are predicted to be targeted into the ER of the host cell, resulting in the alteration in the normal pattern of protein folding in ER.

## 4. Materials and Methods

### 4.1. Proteins and Prediction Analysis

The whole proteome of *M. hominis* comprising 563 proteins was selected for the prediction of proteins that are targeted into host cells. Computational predictions were used for the analysis of proteins targeted into the ER of host cells. Complete data were collected after the prediction analysis to predict implications of ER-targeting proteins in prostate cancer etiology.

### 4.2. Choice of Protein Database for M. hominis

We used the UniProt database (www.uniprot.org) to select the specific strain of *M. hominis*. The UniProt database is considered as a complete resource for the sequence of proteins and data annotation. This database was prepared with the combination of PIR database, Swiss-Prot, and TrEMBL activities [48], and is a collection of huge data with respect to *M. hominis* and its subcellular location, as described in Swiss-Prot/TrEMBL or PIR-PSD [49,50]. This database has two proteomes related to *M. hominis* strains, namely, ATCC-23114/PG21 and ATCC-27545 [51]. All *M. hominis* proteins of ATCC-27545 strain were used for the in silico analysis of the subcellular proteins targeted into host cells by using predictor cNLS mapper (Tsuruoka, Japan). Furthermore, Hum-mPLoc 3.0 predictor (Shanghai, China) was implicated to predict the possible location of ER-targeting *M. hominis* proteins in host cells.

### 4.3. Prediction of the Subcellular Localization by cNLS Mapper

Proteins targeted in various organelles of host cells were predicted using cNLS mapper [44]. Whole protein sequence of *M. hominis* was utilized for the analysis of monopartite and bipartite NLSs. The precise cNLS mapper cutoff values were 8–10, 1–2, 5–3, and 7–8, that were used to predict the localization of proteins in nucleus, cytoplasm, both cytoplasm and nucleus, and partially nucleus, respectively, as defined in the literature of cNLS. The values of monopartite and bipartite NLSs (basic amino acid stretches) were analyzed using the cNLS mapper in whole protein sequence of *M. hominis*. These basic stretches mediate binding of NLS to importin-α transport receptor, and this complex binds to importin β, through which a specific protein localizes to the nucleus. cNLS mapper detects contribution of each residue in NLS and predicts NLS activity, which is suggested to give more accurate prediction performance [44].

### 4.4. Prediction of ER Localization in M. hominis Proteins Using Hum-Mploc 3.0

Hum-mPLoc 3.0 was utilized to determine *M. hominis* protein localization in the ER and covered about 12 different human subcellular compartments. The protein sequences have showed multiview complementary features, i.e., peptide-based functional domains, context vocabulary annotation-based gene ontology, and amino acid residue-based statistical features, as most of the existing predictors to determine the subcellular targeting of human proteins are limited with unique location site. To prevail this barrier, Hum-mPLoc, a new ensemble classifier [47], was established and used for cases with multiple location sites. The predictor Hum-mPLoc is accessible generously by researchers from the web server at http://202.120.37.186/bioinf/hum-multi. This predictor has been employed to predict various human protein entries in Swiss-Prot database which do not have subcellular location annotations or are interpreted as “uncertain.” Hum-mPLoc predictor may analyze the possible targeting of specific protein in three kingdoms, namely, plants, fungi, and animals. In the current research work, we determined proteins targeting in the ER of host cells using *M. hominis* proteins sequence as the query.

## 5. Conclusions

Protein homeostasis/proteostasis, is very crucial for viability and health of cells [33]. Alteration in proteostasis has been connected with the growth of many disorders, including cancer, which is considered as the most challenging disease of the current era [31]. Among different virulence aspects, various bacterial protein toxins that are somehow associated to the progression of various types of cancer have been the possible targets or markers for the management of cancer. The possible connection between *M. hominis* infection and risk of prostate cancer has gained attention in the past few years, but no detailed information is available. Here we predicted the connection between *M. hominis* infection and prostate cancer, and found that the intercellular *M. hominis* infection in host cells acts as a potential factor in prostate cancer etiology, owing to the accumulation of several *M. hominis* proteins in the ER of host cells. ER is an important organelle of eukaryotic cells involved in the regulation of secretory pathways, and release and storage of calcium. Misfolded proteins in ER cause ER stress through their accumulation and stimulation of UPR. ER stress and UPR activation are associated with the progression of different types of diseases in human, including different types of cancer. The present research work paves way for the analysis of the potential involvement of specific strain of *M. hominis* in prostate cancer.

## Figures and Tables

**Table 1:** Computational prediction of *M. hominis* proteins targeted to the endoplasmic reticulum (ER) of host cells and their relation to all proteins with a similar NLS.

NLS	NLS Cutoff	Number of Proteins Targeting ER	Total Number of Proteins in This Range	Percentage
Monopartite NLS	0–3.0	15	509	2.94
	3.0–5.0	3	32	9.3
	5.0–8.0	1	16	6.25
	>8.0	0	6	0
Bipartite NLS	0–3.0	6	76	7.89
	3.0–5.0	11	290	3.79
	5.0–8.0	2	194	1.03
	>8.0	0	3	0

**Table 2:** Computational prediction of *Mycoplasma hominis* proteins targeted to ER of host cells and their relation to all proteins with similar molecular weight.

Molecular Weight	Number of Proteins Targeting to ER	Total Number of Proteins	Percentage
0–20 kDa	3	128	2.34
20–40 kDa	10	205	4.87
40–60 kDa	4	109	3.66
60–80 kDa	1	61	1.63
>80 kDa	1	60	1.66

**Table 3:** Computational prediction of *Mycoplasma hominis* proteins targeting to ER of host cells and their relation to all proteins with similar pI value.

Range of pI Value	Number of Proteins Targeting to ER	Total Number of Proteins	Percentage
3.0–5.0	0	25	0
5.0–6.0	1	105	0.95
6.0–7.0	0	68	0
7.0–8.0	0	35	0
8.0–9.0	2	109	1.83
9.0–10.0	12	181	6.62
10.0–11.0	4	33	12.12
12.0–13.0	0	7	0

**Table 4:** Details of *Mycoplasma hominis* proteins targeted to ER of host cells with their functions.

Accession Number	Protein Name	Function in Bacteria	Protein Existence	pI	Molecular Weight	NLS Mapper		Hum-mPLoc 3.0
						Monopartite	Bipartite	
A0A097NT54	Uncharacterized protein	Unknown	Protein predicted	10.17	13,450	0	22	Endoplasmic reticulum
A0A097NSU8	Uncharacterized protein	Unknown	Protein predicted	5.45	36,963	0	3.8	Endoplasmic reticulum
A0A097NTC6	Lipoprotein signal peptidase (EC 3.4.23.36)	Aspartic-type endopeptidase activity.	Protein inferred from homology	8.44	23,620	0	3.3	Endoplasmic reticulum
A0A097NTZ8	Cation transporting P-type ATPase (EC 3.6.3.8)	ATP binding, calcium-transporting ATPase activity, and metal ion binding activity.	Protein inferred from homology	8.56	107,148	2	6.1	Endoplasmic reticulum
A0A097NT87	Protein translocase subunit SecY	Intracellular protein transmembrane transport activity, protein transport activity hrough the Sec complex	Protein inferred from homology	9.51	54,926	6	3.9	Endoplasmic reticulum
A0A097NTV1	Membrane protein	Unknown	Protein predicted	9.54	25,294	0	5.5	Endoplasmic reticulum
A0A097NTF0	Prolipoprotein diacylglyceryl transferase (EC 2.4.99.-)	Phosphatidylglycerol-prolipoprotein diacylglyceryl transferase activity and involved in lipoprotein biosynthetic process.	Protein inferred from homology	9.56	35,944	0	3.8	Endoplasmic reticulum
A0A097NSJ5	Potassium transporter KtrB	Cation transmembrane transporter activity.	Protein predicted	9.6	58,434	0	3	Endoplasmic reticulum
A0A097NT10	ComEC/Rec2-related protein	Unknown	Protein predicted	9.65	54,059	0	4.4	Endoplasmic reticulum
A0A097NSI4	Uncharacterized protein	Unknown	Protein predicted	9.66	38,283	0	3.3	Endoplasmic reticulum
A0A097NSM2	Membrane protein	Unknown	Protein predicted	9.72	33,519	0	3.4	Endoplasmic reticulum
A0A097NSJ8	1-acyl-sn-glycerol-3-phosphate acyltransferase (EC 2.3.1.51)	1-acylglycerol-3-phosphate *O*-acyltransferase activity.	Protein predicted	9.79	31,034	0	4.7	Endoplasmic reticulum
A0A097NT14	Cobalt ABC transporter permease	Unknown	Protein predicted	9.85	35,998	5	4.4	Endoplasmic reticulum
A0A097NTJ8	ABC transporter permease	Transporter activity.	Protein inferred from homology	9.86	65,590	0	2.9	Endoplasmic reticulum
A0A097NTE8	Uncharacterized protein	Unknown	Protein predicted	9.9	57,701	0	5	Endoplasmic reticulum
A0A097NSN9	Uncharacterized protein	Unknown	Protein predicted	9.97	14,493	0	2.6	Endoplasmic reticulum
A0A097NSG1	Uncharacterized protein	Unknown	Protein predicted	10.1	17,661	4	2.6	Endoplasmic reticulum
A0A097NTR2	Membrane protein	Transporter activity.	Protein predicted	10.19	33,456	4	3.2	Endoplasmic reticulum
A0A097NT45	Membrane protein	Unknown	Protein predicted	10.47	20,769	0	2.5	Endoplasmic reticulum
